# Fluid resuscitation with preventive peritoneal dialysis attenuates crush injury-related acute kidney injury and improves survival outcome

**DOI:** 10.1186/s13049-019-0644-0

**Published:** 2019-07-18

**Authors:** Xian-Long Zhou, Shao-Zhou Ni, Dan Xiong, Xue-Qi Cheng, Peng Xu, Yan Zhao

**Affiliations:** 1 Emergency Center, Zhongnan Hospital of Wuhan University, 169 Donghu Road, Wuhan, 430071 Hubei China; 2 Emergency Department, Xiangyang No.1 People’s Hospital, Hubei University of Medicine, 15 Jiefang Road, Xiangyang, 441000 Hubei China

**Keywords:** Crush injury, Peritoneal dialysis, Massive fluid resuscitation, Acute kidney injury, Survival outcome

## Abstract

**Background:**

In-hospital renal replacement therapy (RRT) is widely used for the treatments of acute kidney injury (AKI) in crush injury (CI) victims. This study was designed to investigate whether preventive peritoneal dialysis (PPD) is useful for renal protection in CI.

**Methods:**

Animals received hindlimb compressions for 6 h to induce CI. Then, animals were untreated or treated with PPD and/or massive fluid resuscitation (MFR) for 8 h since the onset of compression release. Blood and renal tissue samples were collected at various time points for biological and morphological analysis.

**Results:**

PPD attenuated lactic acidosis and reduced serum K^+^ and myoglobin levels in CI animals. In addition, PPD was effective in removing blood urea nitrogen (BUN) and creatinine, and reduced renal expressions of neutrophil gelatinase-associated lipocalin (NGAL). The combination of PPD and MFR furtherly attenuated AKI with significantly decreased histological scores (*p* = 0.037) and reduced NGAL expressions (*p* = 0.0002) as compared with the MFR group. Moreover, MFR + PPD group had a significantly higher survival rate than that in the MFR and the PPD groups (*p* < 0.05, respectively).

**Conclusion:**

The use of PPD at the onset of compression release is beneficial for renal protection and survival outcome in a rabbit model of CI.

**Electronic supplementary material:**

The online version of this article (10.1186/s13049-019-0644-0) contains supplementary material, which is available to authorized users.

## Background

Crush injury (CI) causes rhabdomyolysis and subsequent acute kidney injury (AKI) [1], which is one of the few life-threatening complications of CI that can be prevented [2]. Delay in fluid resuscitation for more than 6 h after CI significantly increases the risk of developing AKI [3]. In many crush casualties, AKI can be prevented by adequate fluid resuscitation, while those who develop acute renal failure (ARF) require extensive treatments and possibly dialysis [4–6]. Currently, fluid resuscitation is considered the first choice of treatment for CI victims. However, the CI-induced AKI cannot be totally prevented; some of the victims who receive early fluid resuscitation still develop AKI after CI [7]. Once severe AKI occurs, different types of in-hospital renal replacement therapy (RRT) can be applied. RRT modalities, including continuous RRT, intermittent hemodialysis (IHD) and peritoneal dialysis have been used in treating CI-induced AKI [8]. Currently, IHD has been suggested as the first choice for crush victims after consideration of medical and logistic assets, and peritoneal dialysis may be preferable for small children during disaster [9]. Generally, RRT should be initiated after the consideration of significant volume overload or solute biochemical imbalance. However, to the best of our knowledge, it has not yet been reported whether preventive RRT is beneficial for CI victims. RRT requires specialist equipment, electricity, and a water supply. However, the shortage of materials is common in the event of large-scale disasters, such as earthquakes. Therefore, regular RRT, especially hemodialysis, is difficult due to loss of lifelines and the damages to faculties. Unlike other RRT modalities, peritoneal dialysis can be rapidly started at the site of the accident with simple materials. Therefore, we designed this study to investigate whether PPD initiated at the onset of compression release was able to prevent CI victims against AKI and to improve the short-term survival.

## Methods

### Animals

Eighty, male specific pathogen-free New Zealand white rabbits weighing about 1.5–2.0 kg were purchased from the Wuhan University Center for Animal Experiment/Animal Biosafety Level III laboratory (ABSL-III lab) of Wuhan University (Wuhan, Hubei, China). Animal experiments were performed between August 2016 and March 2018. Animals were kept in cages under controlled conditions (temperature: 25 ± 2 °C; relative humidity: 50 ± 5%) with a 12:12 light-dark cycle. The water and food were provided ad libitum.

### Study protocol

**Fig. 1:**
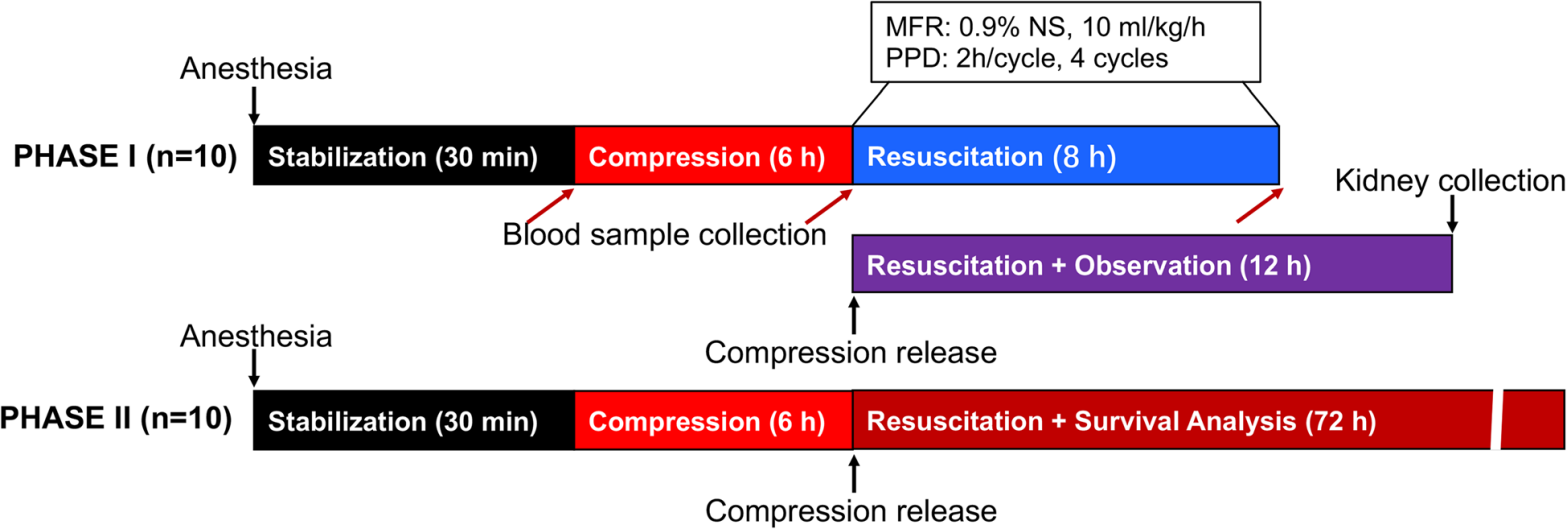
Study protocol. Phase I (*n* = 10): animal study for biochemical and morphological analysis; blood samples were collected at the time prior to compression onset (*Baseline*), the time just after compression release (*Compression*), and the end of resuscitation (*Resuscitation, n* ≥ 5). Kidneys were collected at 12 h after compression release (*n* ≥ 3). Surgical procedure of peritoneal dialysis was performed at 1 h before compression release. Phase II (n = 10): survival analysis without sample collections. In each group, 10 animals were assigned to a 72-h survival observation. MFR = massive fluid resuscitation; PPD = protective peritoneal dialysis; NS = normal saline

Anesthetized and cannulated animals were stabilized for 30 min. Then, animals were randomly assigned into the following 4 groups: a compression group (Control, *n* = 20), a massive fluid resuscitation group (MFR, n = 20), a preventive peritoneal dialysis group (PPD, n = 20) and a massive fluid resuscitation combined with preventive peritoneal dialysis group (MFR + PPD, n = 20). Animals received continuous compression on both hindlimbs for 6 h to create CI. After compression release, animals in the Control group received all the surgical procedures for the preparation of peritoneal dialysis (sham operation) without any treatments. In the MFR group, rabbits received sham operation and fluid infusion with 0.9% saline at a rate of 10 ml/h/kg for 8 h. In a preliminary study, we evaluated the potent usefulness of 3 different strengths of glucose dialysate (i.e., 1.5, 2.5, and 4.25%) for the treatment of CI rabbits. A 72-h observation showed that 2.5% (but not 1.5 and 4.25%) glucose dialysate was able to increase the survival rate of CI animals. Finally, we chose 2.5% glucose dialysate for PPD in the present study. Animals in the PPD group were on peritoneal dialysis for 8 h (20 ml/kg body weight; EPD4–1989 (Ca^2+^: 1.25 mmol/L), Baxter, Shanghai, China) after compression release. Animals in the MFR + PPD group were treated with peritoneal dialysis and fluid infusion at the same time. Peritoneal dialysis was performed for 4 cycles (2 h/cycle) after compression release. Then, dialysis fluid in abdominal cavity was collected via dialysis catheter. Blood samples were collected for biochemical analysis at various time points. The kidney samples were collected at 12 h after compression release. Another 10 rabbits in each group were kept for survival analysis without any sample collections. The study protocol is summarized in Fig. 1.

### Crush injury model

The rabbit CI model was established as we previously described [10]. In detail, animals were anesthetized via intraperitoneal injection of pentobarbital sodium (Amresco, Cleveland, OH; 50 mg/kg body weight) and were fixed in a supine position on a warming blanket. Rabbits were cannulated in the right carotid artery and the left external jugular vein. Arterial blood pressure was monitored by a monitoring system (BL-420F, TaiMeng, Chengdu, China) through the right carotid artery, and fluid administration was applied through the left external jugular vein. Then, animals received continuous compression (10 kg/kg body weight for each hindlimb) on both hindlimbs for 6 h using an apparatus that we’ve already developed.

### Peritoneal dialysis

The surgical procedure of rabbit peritoneal dialysis was performed at 1 h before compression release as previously described [11]. Briefly, animals were prepped and draped on the abdomen and positioned in dorsal recumbency. Then, the abdomen was sterilized with 0.5% iodophor, and a stab incision was made with the scalpel blade a few centimeters lateral to the umbilicus after a local anesthetic with 2% lidocaine solution. A 14-gauge over-the-needle intravenous catheter with stylet was entered into the incision site and tunneled 1.0 cm. The stylet was removed, and a jugular catheter placement guide wire was threaded through the intravenous catheter and into the abdomen. The entry hole was enlarged with a dilator, and a wound drainage tube was threaded over the guide wire and fed into the abdomen. Then, the drainage tube was fixed to the skin at the exit site and covered with sterile gauze. The free end of the catheter (wound drainage tube) was attached to a Y-set tubing for connection to sterile collection system and dialysate solution. To minimize the effect on respiration and the presence of leakage from the catheter site, animals received PPD with 2.5% glucose dialysate at small volume (20 ml/kg body weight, EPD4–1989, Baxter, Shanghai, China) [12]. Dialysate solutions were warmed to 38 °C before infusion, and heparin (250 U/L) was added to the dialysate to prevent catheter occlusion [13]. The dialysate solution was infused over a 10-min period, and the solution was allowed remain in the abdomen for approximately 90 min. Then, the dialysate solution was drained via gravity flow over 20–30 min. The drainage of dialysate solution ended when the solution volume was unchanged. Together, the dialysis cycle was repeated every 2 h and performed for 4 cycles. In addition, the dialysis catheter was placed in all animals, including those assigned into the Control and MFR groups. At the end of treatments, catheters were removed gently. The incision site was sutured and covered with gauze after sterilization.

### Blood sample analysis

Blood samples were collected at 3 different time points: prior to the onset of compression (*Baseline*), compression release (*Compression*), and the end of resuscitation (*Resuscitation*). The blood gas analysis and the measurements of serum electrolytes levels were performed using *i-STAT 1Analyzer* (Abbott, Kyoto, Japan). Blood cell counts were measured by an automatic blood cell analyzer, *Pentra MS CRP* (HORIBA Medical, Kyoto, Japan). Myoglobin, CK, BUN and creatinine were detected using a *TBA- 2000FR System* (TOSHIBA, Tokyo, Japan).

### Calculation of removal rate

To test the efficiency of peritoneal dialysis in the clearance of serum K^+^ and myoglobin, we calculated the removal rate as follows: (1) K^+^ removal rate = −[K^+^ levels at the end of resuscitation - K^+^ levels prior to resuscitation/K^+^ levels prior to resuscitation] × 100%; (2) myoglobin removal rate = −[myoglobin levels at the end of resuscitation - myoglobin levels prior to resuscitation/myoglobin levels prior to resuscitation] × 100%; (3) CK removal rate = −[CK activities at the end of resuscitation - CK activities prior to resuscitation/CK activities prior to resuscitation] × 100%.

### Morphological analysis

Morphological analysis of injured kidneys was performed as we previously described [14]. At the end of experiments, animals were killed by blood dropping under anesthesia. Both kidneys were collected and were cut into 4–6 small pieces. Then, kidney samples were washed with cold PBS, and half of the kidney samples were fixed in 10% formalin, embedded in paraffin, cut into sections 4 μm in thickness, and stained with hematoxylin-eosin (H&E). Then, evaluation for each specimen was performed by two different pathologists in a blind manner. Kidney injury was evaluated based on necrosis of renal tubular epithelial cells (RTECs), hydropic degeneration and steatosis of RTECs, protein casts in renal tubules, and damage of the tubular basement membrane. All specimens were scored ranging from 0 (no visible damage) to 10 (maximal damage). Two pathologists reviewed all the specimens independently, and the mean score was documented for each specimen. Histological scores were determined by a third expert only when the differences in histological scores ≥3 between these two pathologists.

### Confocal immunofluorescence microscopy

Confocal studies were performed in a blinded manner on paraffin-embedded renal tissues. Anti-rat NGAL (Abcam, Shanghai, China) antibody was analyzed using immunofluorescence. Nuclei were stained blue with 4′,6-diamidino-2-phenylindole, dihydrochloride (DAPI) (Thermo Scientific, MA, USA). Fluorescence intensities were analyzed in 10 random fields under confocal microscope (FV1000, Olympus, Tokyo, Japan) of each sample and determined using ImageJ software.

### Survival analysis

In each group, ten out of twenty CI animals without sample collections were kept for a 72-h survival analysis. Animals were kept in individual cages under standard conditions. Animals received food and water ad libitum. Animals were checked by one of our researchers at an interval of 6 h.

### Sample size calculation

On the basis of our preliminary trial related to the survival of CI animals [10], we estimated that fluid resuscitation with PPD would result in absolute increases in survival rate (from 30 to 100%). We calculated that it would require at least 9 animals in each group to have a statistical power of 80% to detect the estimated difference under a two-sided alpha level of 0.1. Survival observation was performed for 72 h after compression release. Therefore, those animals underwent sample (especially kidney) collections were unable to be kept for survival follow-up. Finally, we chose to include 20 animals in each group for survival analysis (10 rabbits) and for biochemical and pathological analysis (10 rabbits).

### Statistical analysis

Data were expressed as number, percentage, and mean ± SD. Comparison of means was performed using one-way analysis of variance (ANOVA). Comparisons between 2 groups were performed by unpaired student-*t* test. Removal rates and histological scores of each group were analyzed using the Steel-Dwass test followed by the Kruskal-Wallis test. The Kaplan-Meier method was applied for survival analysis, and comparison between groups was performed using the log-rank test. Statistical analysis was performed using *R* for Mac OS. A 2-tailed *p* value less than 0.05 was considered as significant.

## Results

### Systemic responses to CI

Our results showed that CI induced systemic disturbance. At *Compression*, CI induced significant decreases in base excess (BE) and significant increases in serum lactate levels as compared to baselines (*p* < 0.05, respectively). PaO_2_ in all groups were increased at *Resuscitation* as compared to baselines, whereas PaCO_2_ levels were decreased (*p* < 0.05, respectively). Importantly, lactate levels were significantly decreased in the PPD and the MFR group as compared with the Control group at *Resuscitation* (*p* < 0.05). The combination of MFR and PPD further decreased lactate levels as compared with the MFR and the PPD group (*p* < 0.05, respectively). However, there were no significant differences in lactate levels between the PPD and the MFR group. Results are summarized in Table 1. Blood cell counts and general characteristics of animals are summarized in Additional file 1: Table S1 and Table S2.

**Table 1:** Arterial blood gases and lactate (mean ± SD)

	Group	Baseline	Compression	Resuscitation
pH	Con	7.34 ± 0.03	7.44 ± 0.05^††^	7.30 ± 0.15^†††**^
	MFR	7.35 ± 0.03	7.43 ± 0.02^†^	7.38 ± 0.06^#^
	PPD	7.34 ± 0.02	7.42 ± 0.04^††^	7.42 ± 0.05^†##^
	MFR + PPD	7.35 ± 0.03	7.42 ± 0.03^†^	7.40 ± 0.03^##^
BE (mmol/L)	Con	1.3 ± 1.2	0.5 ± 1.3^†^	−4.8 ± 1.6^†††***^
	MFR	1.8 ± 2.0	1.0 ± 0.8^†^	−1.2 ± 1.4^††###**^
	PPD	1.5 ± 1.2	0.7 ± 1.2^†^	−1.5 ± 1.7^††###**^
	MFR + PPD	1.7 ± 1.0	0.9 ± 0.7^†^	−1.2 ± 0.9^††###**^
PaCO_2_ (mmHg)	Con	48 ± 9	36 ± 8^††^	28 ± 6^†††**^
	MFR	50 ± 11	42 ± 14^†^	28 ± 5^†††**^
	PPD	50 ± 13	33 ± 12^†††^	32 ± 12^†††^
	MFR + PPD	49 ± 10	37 ± 10^††^	30 ± 10^†††*^
PaO_2_ (mmHg)	Con	91 ± 9	92 ± 5	122 ± 14^†††***^
	MFR	89 ± 12	93 ± 7	112 ± 20^†††#**^
	PPD	91 ± 13	93 ± 9	112 ± 17^†††#**^
	MFR + PPD	88 ± 10	92 ± 7	114 ± 19^†††#**^
Lactate (mmol/L)	Con	1.0 ± 0.4	2.5 ± 0.9^†††^	5.7 ± 1.2^†††**^
	MFR	0.9 ± 0.3	2.1 ± 0.4^†††^	2.8 ± 0.5^†††*##^
	PPD	1.1 ± 0.4	2.1 ± 0.4^††^	2.2 ± 1.0^†††###^
	MFR + PPD	0.8 ± 0.3	1.6 ± 0.5^††^	1.9 ± 0.6^†††###^

*BE* base excess, *Con* control group, *MFR* massive fluid resuscitation, *PPD* preventive peritoneal dialysis. In each group, number of animals ≥5 at all time points. ^#^ *p* < 0.05, ^##^ *p* < 0.01, ^###^ *p* < 0.001 vs. Control group; ^†^ *p* < 0.05, ^††^ *p* < 0.01, ^†††^ *p* < 0.001 vs. *Baseline*; ^*^ *p* < 0.05, ^**^ *p* < 0.01, ^***^ *p* < 0.001 vs. *Compression*

### Changes in blood pressures after CI

**Fig. 2:**
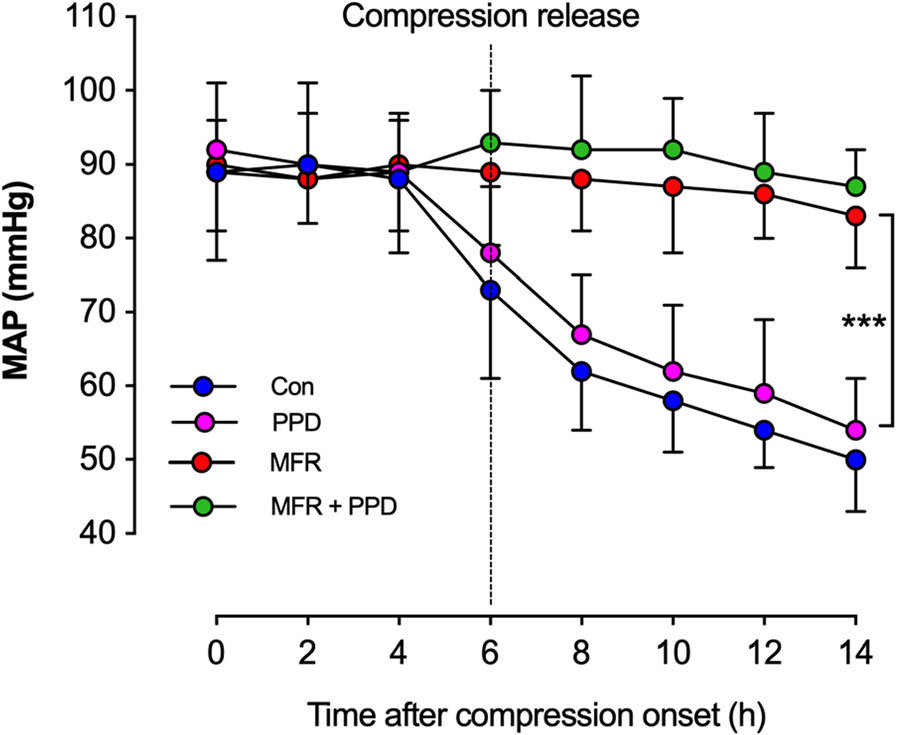
Blood pressure monitoring in all groups. The mean arterial pressure (MAP) was monitored and data were collected with a 2-h interval. Hindlimb compression lasted for 6 h, and the MAP monitoring ended at 8 h after compression release. In each group, the number of animals was ≥5 at all time points. Con = control group; MFR = massive fluid resuscitation; PPD = preventive peritoneal dialysis. ^***^ *p* < 0.001

As detailed in Fig. 2, we observed a sharp decrease in MAP after compression release in the Control and the PPD groups, but not in the MFR and the MFR + PPD groups. Although hypotension has likely been attenuated by PPD, the differences in MAP between the PPD and the Control group were insignificant (*p* > 0.05).

### Clearance of circulating K^+^, myoglobin, and CK in CI animals

**Fig. 3:**
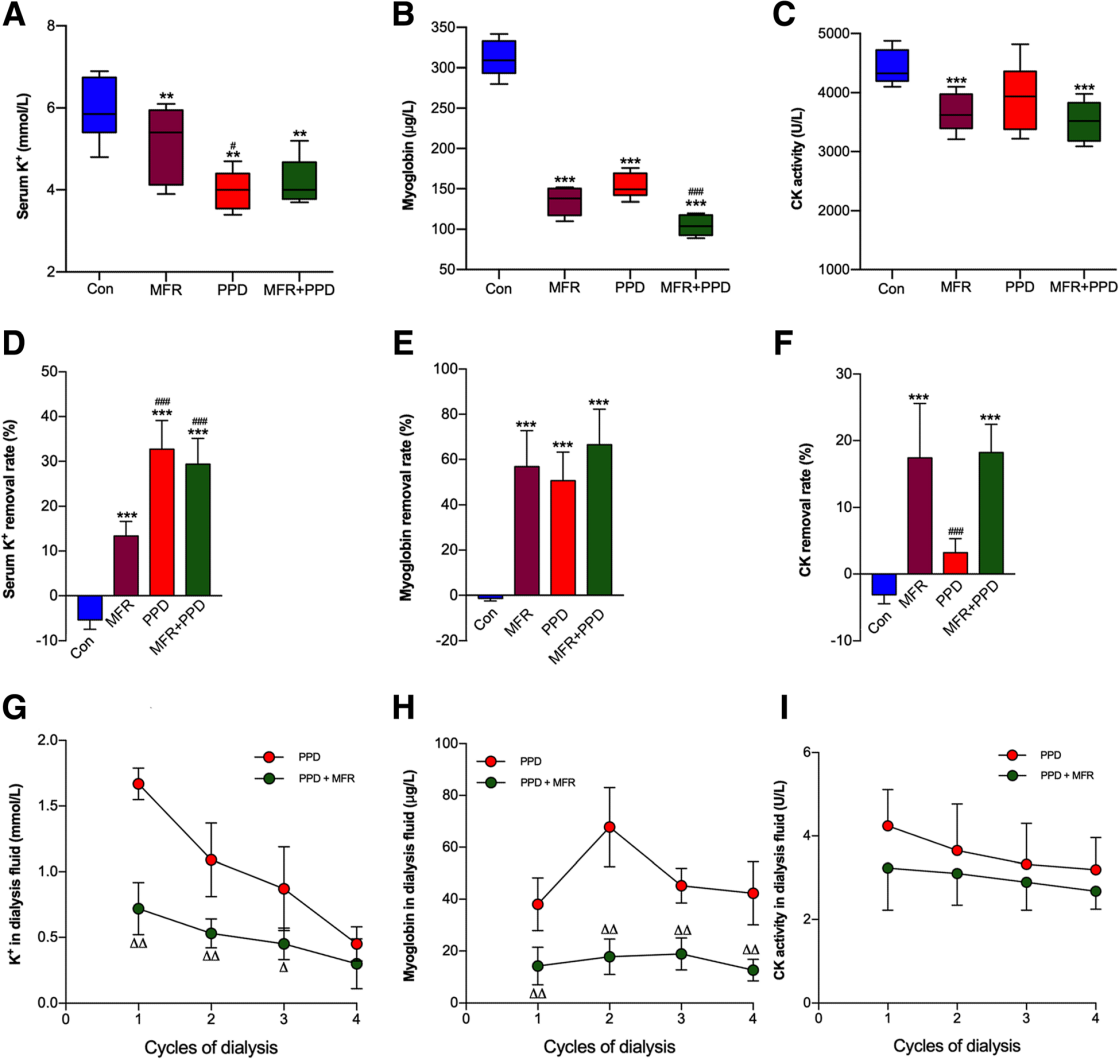
Effects of MFR and PPD on CI-induced rhabdomyolysis. At the end of resuscitation, we measured serum K^+^ levels (**a**), myoglobin concentrations (**b**), and CK activities (**c**) to evaluate rhabdomyolysis. To evaluate the clearance ability of different interventions, removal rates of serum K^+^ (**d**), myoglobin (**e**) and CK (**f**) were calculated. Dialysis fluids were collected for the measurements of K^+^ (**g**), myoglobin (**h**) levels and CK activities (**i**). In each group, the number of animals was ≥5 at all time points. Con = control group; MFR = massive fluid resuscitation; PPD = preventive peritoneal dialysis. In each group, number of animals ≥5. ^*^p < 0.05, ^**^ *p* < 0.01, ^***^ *p* < 0.001 vs. CP group; ^#^*p* < 0.05, ^##^*p* < 0.01, ^###^*p* < 0.001 vs. MFR group. ^∇∇^ *p* < 0.01 vs. PPD group

High concentrations of muscle cell contents, including electrolytes, enzymes, and proteins are absorbed into the circulation system when rhabdomyolysis occurs. Serum K^+^ and myoglobin levels were reduced by either MFR or PPD (Fig. 3a, b). However, PPD alone was unable to reduce CK activities in CI animals (Fig. 3c). Compared to MFR, PPD was more efficient for the removal of serum K^+^ as reflected by higher removal rates (Fig. 3d-f). In addition, a certain amount of K^+^ and myoglobin have been determined in dialysis fluids, whereas the CK activities were negligible (Fig. 3g-i). These results suggested that PPD was able to remove serum K^+^ and myoglobin from circulation to the abdomen cavity. However, the effects of PPD on the clearance of CK have not been observed in this study.

### PPD prevents animals from CI-induced AKI

**Fig. 4:**
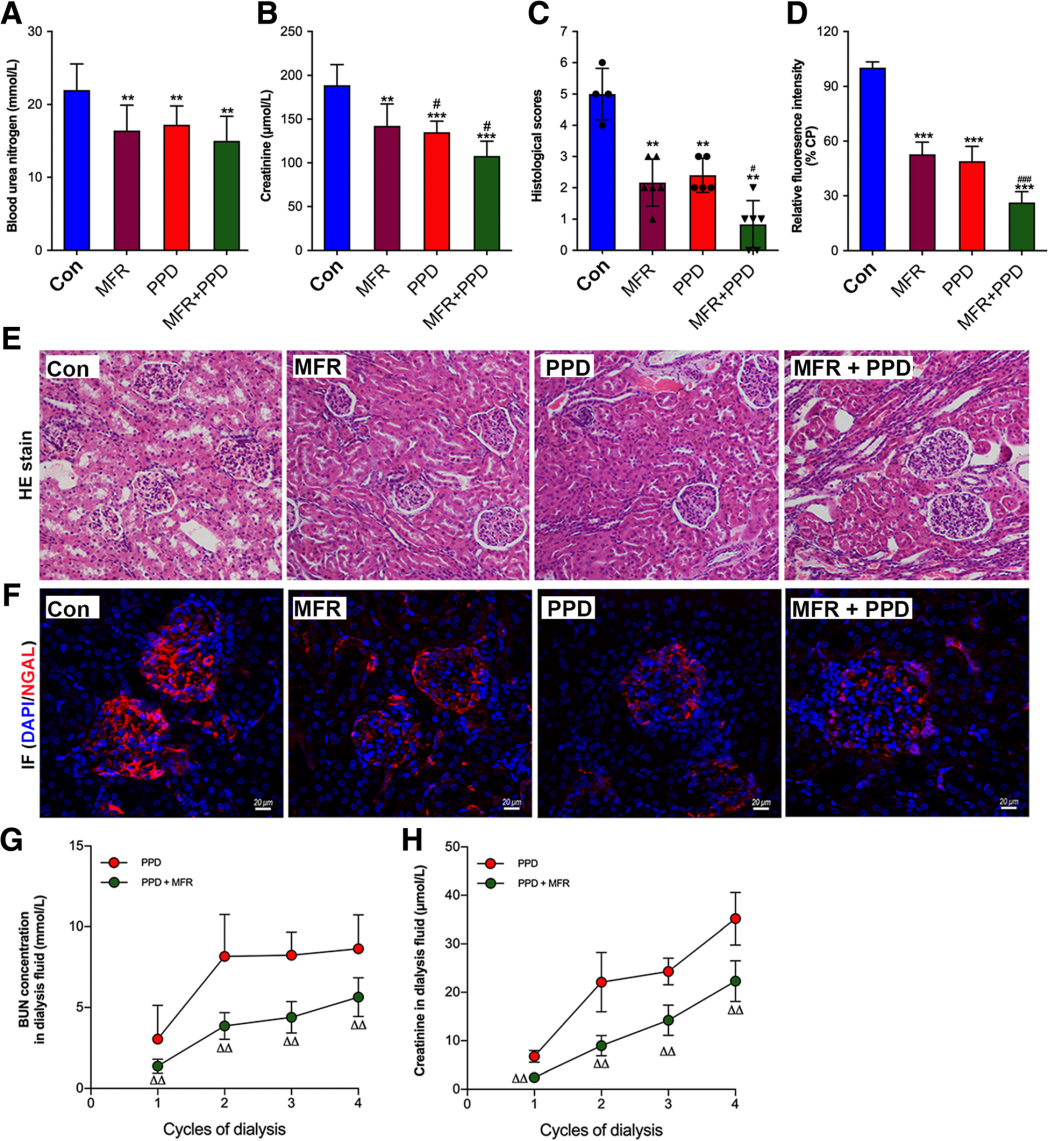
Evaluation of kidney injury. To evaluate kidney injury, BUN (**a**) and creatinine (**b**) were determined at the end of resuscitation. In addition, specimens of renal tissues were scored (**c**) after HE stain (**e**, 200×). Immunofluorescence stain of renal NGAL were performed to evaluate acute kidney injury (**d**, **f**). BUN (**g**) and creatinine (**h**) were also detectable in dialysis solutions collected from abdominal cavity. BUN = blood urea nitrogen; Con = control group; MFR = volume resuscitation; PPD = preventive peritoneal dialysis; NGAL = neutrophil gelatinase-associated lipocalin. In each group, number of animals ≥5 for biochemical analysis and number of animals ≥4 for morphological assessments. ^**^p < 0.01, ^***^ *p* < 0.001 vs. CP group; ^#^*p* < 0.05, ^###^*p* < 0.001 vs. MFR group; ^ΔΔ^ *p* < 0.01 vs. PPD group

We found that blood creatinine and BUN levels were significantly decreased in the PPD and MFR groups as compared with the Control group at the end of resuscitation (*p* < 0.05, respectively) (Fig. 4a, b). As seen in Fig. 4c, e, h&e staining demonstrated numerous protein casts in renal tubules and renal tubular epithelial cell damage in the CI animals, and histological scores were significantly lower in the MFR and the PPD group than that in the Control group (*p* < 0.01, respectively). However, there were no significant differences in histological scores between the MFR and the PPD group (*p* < 0.05). In addition, histological scores were furtherly decreased in the MFR + PPD group as compared with the MFR group (*p* < 0.05). In this study, renal expressions of neutrophil gelatinase-associated lipocalin (NGAL) were determined as an early marker of AKI. As seen in Fig. 4d, f, fluorescence intensities of NGAL in the MFR and PPD groups were significantly decreased as compared with the Control group, and the combination of MFR and PPD further reduced NGAL expression as reflected by the lowest fluorescence intensity (*p* < 0.01, respectively). In addition, both BUN and creatinine were detectable in dialysis solutions collected from abdominal cavity (Fig. 4g, h).

### Short-term survival observation of injured animals

**Fig. 5:**
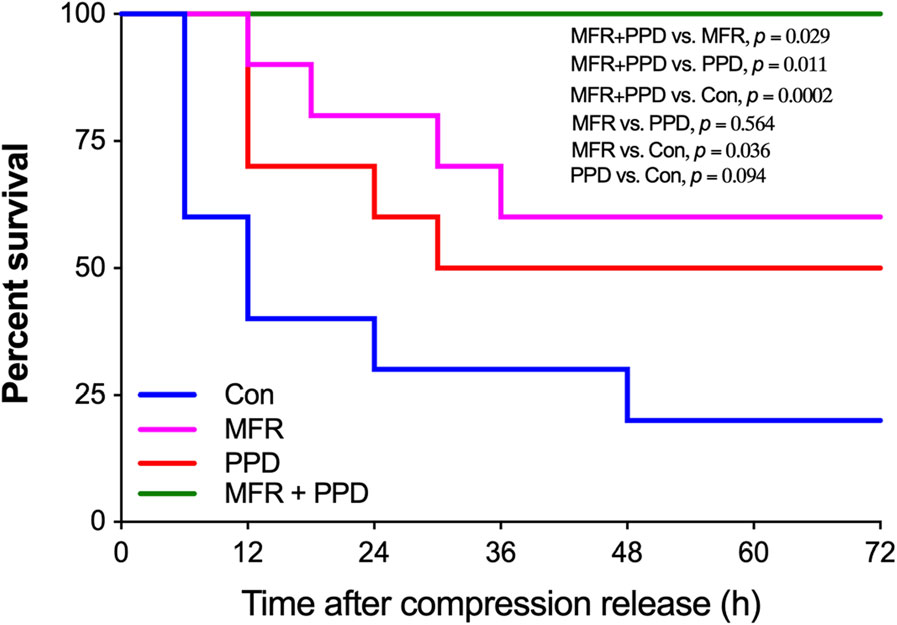
Survival analysis. A 72-h survival observation was carried out in all groups (*n* = 10 in each group). The status of all animals was checked every 6 h during observation. Con = control group; MFR = massive fluid resuscitation; PPD = preventive peritoneal dialysis

Survival analysis is summarized in Fig. 5. The survival rate of animals in the Control, MFR, PPD, and MFR + PPD groups were 20% (2/10), 60% (6/10), 50% (5/10) and 100% (10/10), respectively. There was no significant difference in survival rate between the Control and the PPD groups (*p* = 0.094). However, MFR was able to improve the short-term survival rate of CI animals as compared with the Control group (*p* = 0.036). Moreover, the combination of MFR and PPD further improved the animal’s survival as compared to the MFR group (*p* = 0.029).

## Discussion

The major findings of this study can be summarized as follows: (1) MFR is essential for the correction of CI-induced hypotension, whereas PPD with 2.5% glucose dialysate exerted limited effects on blood pressures in CI animals; (2) the combination of MFR and PPD protected animals against lactic acidosis, rhabdomyolysis and AKI following CI, which finally resulted in an improved survival rate. These results suggested that, in addition to MFR, PPD was beneficial for renal protection in CI rabbits.

The incidence of CI-related ARF varies among different reports. Overall, up to 25% of hospitalized CI victims from disaster appear to be at risk of ARF [15–17]. The impaired kidney blood perfusion, intratubular obstruction by myoglobin, and severe hyperkalemia are the major factors that contribute to the pathogenesis [8]. To increase renal perfusion, the maintenance of blood pressures should be considered first. It has been well demonstrated that fluid resuscitation (10 to 15 ml/kg/h) prior to extrication, or at least in the first 6 h after rescue, is essential for the prevention of AKI [5]. In the present study, we observed that MFR with 0.9% saline at a rate of 10 ml/kg/h was able to correct CI-induced hypotension and to attenuate CI-induced AKI, whereas peritoneal dialysis alone failed to improve blood pressures in CI animals after compression release. These findings indicated that fluid resuscitation is an effective treatment for CI-induced hypotension. In addition, the use of PPD with 2.5% glucose dialysate in the present study exerts no additional effects on dynamics in CI animals.

RRT is generally initiated in CI victims for standard indications, such as volume overload, severe uremia, hyperkalemia, etc [8] Here, instead of performing peritoneal dialysis in a standard protocol, for the first time, we reported the effects of PPD for renal protection in CI rabbits. Our results suggested that PPD initiated at the time of compression release is beneficial for the correction of CI-related hyperkalemia and myoglobinemia. The K^+^ clearance rate achieved by peritoneal dialysis has been shown to be lower than hemodialysis. However, peritoneal dialysis could be the only achievable RRT for K^+^ clearance under some circumstances, such as the shortage of electricity and equipment in large disasters. The normalization of serum K^+^ is the priority because hyperkalemia is an important risk factor of life-threatening conditions, such as cardiac arrest. Recently, *Roseman* et al. demonstrated the potential utility of peritoneal dialysis as a treatment option for severe hyperkalemia patients when HD access is limited [18]. In the present study, we calculated the removal rate of serum K^+^ by MFR and PPD. Our results demonstrated that PPD with 2.5% glucose dialysate was more efficient in removing K^+^ than MFR in CI rabbits. Clinically, peritoneal dialysis patients have normal or low plasma K^+^, probably due to the shift of K^+^ into the intracellular compartment, which is facilitated by low pH and/or by the hyperosmolality of the dialysate [19]. However, in the present study, we observed that PPD attenuated lactic acidosis in CI animals. In addition, analysis of collected dialysis fluids demonstrated a certain amount of K^+^. These results suggested that serum K^+^ was shifted into the abdominal cavity when PPD was applied. The major mechanisms of renal toxicity by myoglobin include renal vasoconstriction, intratubular cast formation and the direct toxicity to tubular cells [20–22]. RRT has also been also applied for circulating myoglobin removal, and there was no significant difference between peritoneal dialysis and IHD for myoglobin removal in end-stage kidney disease patients [23]. In the present study, our results suggested that circulatory myoglobin can be removed by PPD in CI animals, and we detected myoglobin in dialysis fluids collected from the abdomen cavity. CK levels are the most sensitive indicator of rhabdomyolysis, and higher CK levels are associated with greater burden on the kidneys. However, it has been demonstrated that even extremely increased CK has no toxic effects [24]. In this study, the clearance ability of CK by PPD was likely limited as compared with fluid resuscitation. Indeed, peritoneal dialysis is inadequate to remove the large solute loads in patients with rhabdomyolysis-induced renal impairment [25].

Since hyperkalemia and hypermyoglobinemia have been attenuated by MFR + PPD strategy, we next aimed to determine whether MFR + PPD protects kidney against acute injury. Histological analysis demonstrated numerous protein casts in the Control group, and either MFR or PPD markedly reduced the number of protein casts. In addition, we detected renal NGAL expressions as a marker of AKI. NGAL has been found to be one of the most important biomarkers for early detection of AKI [26]. In addition to various inflammatory cells, NGAL is secreted by the tubular lining of epithelial cells in the kidneys [27]. Due to early secretion of NGAL from the nephron, its level increases 48 h before any sensible changes in serum or urine creatinine levels [28]. In the present study, we found that renal NGAL expressions at 12 h after compression release have been reduced by PPD. Collectively, our results demonstrated that the renal protective effects of PPD for CI animals were possibly associated with its ability of myoglobin and K^+^ clearance. Although PPD is useful for the correction of lactic acidosis and for the clearance of K^+^ and myoglobin, it failed to induce significant difference in survival rate as compared with controls (50% vs. 20%). However, the combination of MFR + PPD strategy markedly improved the survival rate of CI animals from 20% (Control group) to 100% within 72 h after compression release. The small number of animals in survival analysis may be a reason. However, the uncorrected hypotension in the PPD group was definitely another contributor to these results.

There are two major limitations in this study. First, we only performed a short-term survival analysis in CI animals. Our experiments suggested that the use of PPD is beneficial for survival outcome, but it is uncertain whether PPD is able to reduce the use of renal replacement therapy and to increase long-term survival rate. However, our previous studies have demonstrated that the majority of deaths occurs within 48 h after compression release [10, 14]. In addition, it is difficult to access the long-term survival outcome in an animal model. Therefore, the survival observation was ended at 72 h after compression release in the present study. Second, although our results support the hypothesis that PPD is beneficial for renal protection in CI animals, the implementation of this strategy in CI patients at the site of the accident seems to be difficult. However, our results remind us that the initiation of RRT at the early stage probably improves the survival outcome of CI victims. Currently, RRT is initiated only when significant volume overload or solute biochemical imbalance occurs. Next, the optimal time to initiate RRT for CI victims needs to be investigated in clinical trials.

## Conclusion

Together, our results suggested that MFR is critical for the hypotension management for CI victims. In addition, PPD was beneficial for the correction of lactic acidosis, rhabdomyolysis, and AKI following CI. Therefore, in addition to MFR, PPD could be used for CI management at the early stage.

## Additional file

Additional file 1: **Table S1.** General characteristics of animals (mean ± SD). **Table S2.** Blood cell counts (mean ± SD). (DOCX 19 kb)

## Data Availability

The data are available from the corresponding author upon request.

## Abbreviations

AKI: Acute kidney injury
ARF: Acute renal failure
CI: Crush injury
IHD: Intermittent hemodialysis
MFR: Massive fluid resuscitation
NGAL: Neutrophil gelatinase-associated lipocalin
PPD: Preventive peritoneal dialysis
