# Supplementary material for: Fluid resuscitation with preventive peritoneal dialysis attenuates crush injury-related acute kidney injury and improves survival outcome
Source: Scand J Trauma Resusc Emerg Med. 2019 Jul 18;27:68. doi: 10.1186/s13049-019-0644-0 (PMC6637650; doi:10.1186/s13049-019-0644-0)
Supplement: Supplementary file 1 — Table S1. General characteristics of animals (mean ± SD). Table S2. Blood cell counts (mean ± SD). (DOCX 19 kb) [file 13049_2019_644_MOESM1_ESM.docx]

**Table S1. General characteristics of animals (mean ± SD)**

| Parameters | Group | Baseline | Compression | Resuscitation |
| --- | --- | --- | --- | --- |
| BW (kg) | CP | 1.7 ± 0.2 | NA | 1.6 ± 0.3 |
|  | MFR | 1.8 ± 0.3 | NA | 1.7 ± 0.2 |
|  | PPD | 1.8 ± 0.4 | NA | 1.7 ± 0.3 |
|  | MFR + PPD | 1.7 ± 0.2 | NA | 1.8 ± 0.4 |
| HR (bpm) | CP | 267 ± 22 | 273 ± 21 | 298 ± 42^**^ |
|  | MFR | 275 ± 31 | 266 ± 32 | 278 ± 36^##^ |
|  | PPD | 270 ± 27 | 277 ± 40 | 289 ± 31^**^ |
|  | MFR + PPD | 272 ± 39 | 268 ± 32 | 270 ± 27^##^ |
| RT (℃) | CP | 38.3 ± 0.3 | 38.2 ± 0.5 | 38.1 ± 0.3 |
|  | MFR | 37.9 ± 0.4 | 38.2 ± 0.4 | 38.3 ± 0.5 |
|  | PPD | 38.4 ± 0.3 | 38.1 ± 0.4 | 38.0 ± 0.6 |
|  | MFR + PPD | 38.3 ± 0.2 | 38.2 ± 0.3 | 37.9 ± 0.3 |

Data were expressed as mean ± SD; BW = body weight; HR = heart rate; RT = rectal temperature; CP = compression group; MFR = massive fluid resuscitation; PPD = preventive peritoneal dialysis. In each group, number of animals ≥ 5. ^#^ *p* < 0.05, ^##^*p* < 0.01 vs. CP group; ^*^ *p* < 0.05, ^**^*p* < 0.01 vs. baseline.

**Table S2. Blood cell counts (mean ± SD)**

|  | Group | Baseline | Compression | Resuscitation |
| --- | --- | --- | --- | --- |
| RBC (10^12^/L) | CP | 4.3 ± 0.2 | 4.2 ± 0.2 | 4.5 ± 0.1 |
|  | MFR | 4.3 ± 0.2 | 4.2 ± 0.1 | 4.1 ± 0.2^##^ |
|  | PPD | 4.2 ± 0.1 | 4.2 ± 0.2 | 4.3 ± 0.2 |
|  | MFR + PPD | 4.2 ± 0.2 | 4.1 ± 0.1 | 4.0 ± 0.2^##^ |
| Neutrophil (10^9^/L) | CP | 1.7 ± 0.2 | 1.8 ± 0.1 | 1.8 ± 0.2 |
|  | MFR | 1.8 ± 0.2 | 1.7 ± 0.2 | 1.8 ± 0.2 |
|  | PPD | 1.7 ± 0.1 | 1.7 ± 0.1 | 1.7 ± 0.2 |
|  | MFR + PPD | 1.7 ± 0.1 | 1.8 ± 0.2 | 1.7 ± 0.1 |
| Platelet (10^9^/L) | CP | 122 ± 27 | 120 ± 18 | 122 ± 25 |
|  | MFR | 118 ± 35 | 120 ± 23 | 124 ± 35 |
|  | PPD | 124 ± 22 | 122 ± 25 | 123 ± 26 |
|  | MFR + PPD | 125 ± 24 | 126 ± 32 | 122 ± 23 |
| Hemoglobin (g/L) | CP | 101 ± 21 | 102 ± 19 | 112 ± 24 |
|  | MFR | 107 ± 18 | 104 ± 17 | 101 ± 27^#^ |
|  | PPD | 104 ± 21 | 104 ± 20 | 108 ± 19 |
|  | MFR + PPD | 105 ± 22 | 103 ± 24 | 100 ± 29^#^ |

Data were expressed as mean ± SD; RBC = red blood cell; CP = compression group; MFR = massive fluid resuscitation; PPD = preventive peritoneal dialysis. In each group, number of animals ≥ 5. ^#^ *p* < 0.05, ^##^*p* < 0.01 vs. CP group.
